# Identification of an epithelial-mesenchymal transition-related lncRNA prognostic signature for patients with glioblastoma

**DOI:** 10.1038/s41598-021-03213-y

**Published:** 2021-12-08

**Authors:** XinJie Yang, Sha Niu, JiaQiang Liu, Jincheng Fang, ZeYu Wu, Shizhang Ling, GuangFu Di, XiaoChun Jiang

**Affiliations:** 1 Department of Neurosurgery, The Translational Research Institute for Neurological Disorders, the First Affiliated Hospital (Yijishan Hospital), Wannan Medical College, Wuhu, Anhui China; 2 Department of Neurosurgery, the First Affiliated Hospital of Wannan Medical College (Yijishan Hospital), Wannan Medical College, Wuhu, Anhui China

**Keywords:** Cell signalling, Nuclear transport, Protein transport

## Abstract

Glioblastoma (GBM) is a strikingly heterogeneous and lethal brain tumor with very poor prognosis. LncRNAs play critical roles in the tumorigenesis of GBM through regulation of various cancer-related genes and signaling pathways. Here, we focused on the essential role of EMT and identified 78 upregulated EMT-related genes in GBM through differential expression analysis and Gene set enrichment analysis (GSEA). A total of 301 EMT-related lncRNAs were confirmed in GBM through Spearman correlation analysis and a prognostic signature consisting of seven EMT-related lncRNAs (AC012615.1, H19, LINC00609, LINC00634, POM121L9P, SNHG11, and USP32P3) was established by univariate and multivariate Cox regression analyses. Significantly, Kaplan–Meier analysis and receiver-operating-characteristic (ROC) curve validated the accuracy and efficiency of the signature to be satisfactory. Quantitative real-time (qRT)-PCR assay demonstrated the expression alterations of the seven lncRNAs between normal glial and glioma cell lines. Functional enrichment analysis revealed multiple EMT and metastasis-related pathways were associated with the EMT-related lncRNA prognostic signature. In addition, we observed the degree of immune cell infiltration and immune responses were significantly increased in high-risk subgroup compared with low-risk subgroup. In conclusion, we established an effective and robust EMT-related lncRNA signature which was expected to predict the prognosis and immunotherapy response for GBM patients.

## Introduction

Glioblastoma (GBM), originating from astrocytes or oligodendrocytes precursor cells, is the most common and aggressive primary brain malignancy in adults and accounts for about 80% of malignant gliomas^1^. With the characteristics of high morbidity, high recurrence and high mortality, GBM gravely endangers human health and threaten the lives of patients^2,3^. Currently, clinical treatments for GBM patients includes neurosurgery, radiotherapy, chemotherapy, molecular targeted therapy, and immunotherapy^4^. However, the prognosis of GBM patients remains very poor due to its high recurrence and metastasis rates^5^. Statistically, the median survival time of patients with GBM is only approximately 8–15 months even with standard treatment^6^. Therefore, there is an urgent need to explore novel molecular biomarkers for personalized treatment and accurate prognostic assessment of GBM patients.

Long non-coding RNAs (lncRNAs) is a class of transcripts longer than 200 nucleotides with limited protein-coding potential, which were primarily transcribed from different regions of the genome by RNA polymerase II^7^. Compared with mRNAs, the relative expression levels of lncRNAs are generally lower and exhibit more specific in different cell types, tissues, developmental stages and even diseases. More importantly, a great deal of lncRNAs have been reported to participate in mediation of a variety of biological processes, including development and homeostasis, and the pathogenesis of various diseases^8,9^. LncRNA may regulate gene expression at various levels, including epigenetics, transcription, and post-transcription^10^. Accumulating studies have revealed important roles of lncRNAs in the occurrence and development of GBM. For example, Liu et al. found that the expression of lncRNA SOX2OT was significantly up-regulated in temozolomide-resistant cells and samples from GBM-relapsed patients; and upregulation of SOX2OT was closely associated with poor prognosis of GBM patients. Mechanistically, lncRNA SOX2OT upregulates SOX2 expression and activates Wnt5α/β-catenin signaling pathway, thereby facilitating cell proliferation and temozolomide resistance. These results suggested lncRNA SOX2OT could be used as a prognostic biomarker and a target of temozolomide resistance therapy for GBM patients^11^. Currently, the fast development of lncRNA-targeting therapies has the potential to open new avenues for treating human malignancies including GBM. More importantly, existing studies indicates lncRNA play crucial roles in the occurrence and development of various cancer mainly via modulation of cancer-related genes and signaling pathways. Therefore, it is of great significance to explore lncRNAs involving in critical genes and pathways in progression and aggressiveness of GBM.

In the present study, we identified epithelial-mesenchymal transition (EMT) as one of dominant drivers of GBM through the results of Gene set enrichment analysis (GSEA). EMT refers to the conversion of tumor cells from an epithelial to a mesenchymal-like phenotype. In the process of EMT, tumor cells lose epithelial characteristics, including adherens junctions and apical-basal polarity, and gain migratory and invasive capabilities, which greatly contribute to pathogenesis, progression, and metastasis of tumors. Through overlapping EMT-enriched genes and dysregulated genes in GBM, we obtained 78 upregulated EMT-related genes in GBM. To data, increasing lncRNAs have been demonstrated to participate in the EMT of glioma through various mechanisms. For example, lncRNA H19, XIST, FOXD2-AS1, HOXC-AS2 act as molecular sponges for miRNAs to promote EMT-related genes and thus facilitate EMT, migration and invasion of glioma cells^12–15^. Besides, lncRNA RP11-84E24.3 was reported to directly interact with TFAP2C protein to activate SNAI1 expression and thereby triggering the EMT in glioma cells^16^. Even though, lncRNAs associated with EMT in GBM and their prognostic value remain largely unclarified yet. Therefore, we analyzed the correlation of lncRNA and the 78 EMT-related genes through Spearman correlation analysis and identified 301 lncRNAs which were significantly associated with EMT in GBM. Then we systematically investigated the prognostic value of these 301 EMT-related lncRNAs and constructed an EMT-related lncRNAs prognostic signature for GBM patients. Noteworthy, its prognostic ability was assessed to be satisfactory. Furthermore, we explored the underlying mechanisms of the prognostic signature and found it was significantly correlated with multiple EMT and metastasis-related pathways, and immune activities of GBM.

## Material and methods

### Data acquisition and procession

The gene expression data of HTSeq-FPKM, somatic mutation data (MuTect2 Variant Aggregation and Masking) and clinical phenotype data of The Cancer Genome Atlas (TCGA)-GBM were downloaded from the UCSC Xena database (http://xena.ucsc.edu/). The normalized gene expression data and clinical information of GSE4290 was downloaded from the Gene Expression Omnibus (GEO) using the R package “GEOquery”. Additionally, RNA-seq data of GBM and clinical data from the Chinese Glioma Genome Atlas (CGGA) were downloaded from CGGA data portal (http://www.cgga.org.cn). For TCGA-GBM and CGGA-GBM, the human genome annotation file provided by Ensembl (http://grch37.ensembl.org/) was used to acquire lncRNA and mRNA expression profiles, respectively. TCGA-GBM cohort includes 5 normal tissues and 168 tumor tissues from GBM patients. CGGA-GBM cohorts contains 388 GBM samples. For GSE4290, mRNA expression data of 23 non-tumor tissues and 77 GBM tissues were screened out and annotated by GPL570 platform (Affymetrix Human Genome U133 Plus 2.0 Array).

### Differential expression analysis

R package “limma” was used to performed differential expression analysis to identify differentially expressed gens between two groups. Genes with absolute log2FoldChange (|log2FC|) ≥ 1 and Benjamini-Hochberg-adjusted *P* value (adj. *P*) < 0.05 were considered differentially expressed. Then heatmap of differentially expressed lncRNAs (mRNAs) were generated by R package “pheatmap”. The volcano plot of differentially expressed lncRNAs (mRNAs) was visualized by R package “ggplot2”.

### Gene set enrichment analysis (GSEA)

The Hallmark gene sets in the Molecular Signatures Database v7.4 (MSigDB, https://www.gsea-msigdb.org/) were selected for GSEA to explore key hallmarks of GBM. The mRNA expression data from GSE4290 was utilized to perform GSEA using the R package “clusterProfiler”^17^. Adjusted *P* values (*p* < 0.05) were referenced to select key hallmarks for further analysis.

### Acquisition of EMT-related lncRNAs

Taking the intersection of genes enriched in EMT from GSEA results, upregulated mRNAs in GBM from TCGA, and upregulated mRNAs in GBM from GSE4290 to acquire upregulated EMT-related genes in GBM; Taking the intersection of genes enriched in EMT from GSEA results, downregulated mRNAs in GBM from TCGA, and downregulated mRNAs in GBM from GSE4290 to acquire downregulated EMT-related mRNAs in GBM. Then the correlation of lncRNAs and EMT-related genes in GBM were analyzed by Spearman correlation analysis, with absolute of correlation coefficients |r|> 0.4 and *P* values < 0.05 as cutoff. LncRNAs which were significantly correlated with EMT-related genes were considered as EMT-related lncRNAs in GBM.

### Construction and efficiency evaluation of the EMT-related lncRNA signature

In total, 153 patients of TCGA-GBM cohort with follow-up time > 30 days and complete EMT-related lncRNA expression data were used as training set to establish prognostic signature. 387 GBM patients of CGGA-GBM cohort with follow-up time > 30 days were used as validating set.

Univariate Cox regression analysis was performed to evaluate the correlation of EMT-related lncRNAs and overall survival (OS) of GBM patients. *P* < 0.05 was set as the statistical threshold to identify prognostic EMT-related lncRNAs in GBM. LncRNAs with hazard ratios (HRs) > 1 were considered as risk factors whereas lncRNAs with hazard ratios (HRs) < 1 were deemed as protective factors. Prognostic lncRNAs with expression data in CGGA-GBM cohort were subjected to following multivariate Cox regression analysis. Based on the results of multivariate Cox regression analysis, a prognostic risk score signature was established using a linear combination of the expression levels of EMT-related lncRNAs in the signature multiplied by their corresponding regression coefficients. The risk score of GBM patients is calculated according to the following formula: risk score = EMT-related lncRNA_1_ expression × *β*1 + EMT-related lncRNA_2_ expression × *β*2 + ⋯ + EMT-related lncRNA_*n*_ expression × *βn*. Univariate and multivariate Cox regression analyses were both performed using R package “survival”.

GBM patients were divided into high-risk and low-risk subgroups by the median risk score of TCGA-GBM cohort as the cut-off value. Kaplan–Meier survival analysis was performed by R package of “survival” and “survminer” to evaluate the predictive ability of the EMT-related lncRNA prognostic signature. Besides, the R package “timeROC” was used to draw receiver operating characteristic (ROC) curves at 1-, 3-, and 5-years and calculate corresponding aera under curve (AUC) to validate the prediction efficiency of the EMT-related lncRNA prognostic signature. These analyses were performed for the training dataset (TCGA-GBM) and validation dataset (CGGA-GBM), respectively.

### Cell culture, RNA extraction, and quantitative real-time (qRT)-PCR.

Normal human glial cell line HEB and glioma cell lines (U87 and U251) were provided by American Type Culture Collection (ATCC, USA) and cultured with the Dulbecco’s modified Eagle’s medium (DMEM, Gibco, USA) supplemented with 10% fetal bovine serum (FBS, Gibco, USA) and antibiotics (100 U/ml penicillin and 100 μg/ml streptomycin, Gibco, USA) in the humidified incubator with 5% CO2 under 37 °C. Cells were harvested in the logarithmic growth phase using Trizol (Invitrogen, USA) to extract total cellular RNA according to the standard protocol. Following total RNA were reversed into complementary DNA (cDNA) using the High Capacity cDNA reverse transcription (Thermo Fisher, China). qRT-PCR was conducted using SYBR green mix (FastStart Universal SYBR Green Master, Roche, USA) on the 7500 Real-time PCR System (Applied Biosystems, USA). The relative expression levels of RNA were normalized by GAPDH and calculated using the 2^–ΔΔCT^ method. All experiments were repeated at least three times independently. The Primers used for qRT-PCR were synthesized by Sangon Biotech (China) and all the primer sequences were listed as follows: AC012615.1: F 5’- GGACTCCGCCGTGTCTTCATTG-3’, R 5’-ACCAAATACCCTTTCAGGCTCCTTG-3’; H19: F 5’-TTCAAAGCCTCCACGACTC TGTTTC-3’, R 5’-CGTCTCCACAACTCCAACCAGTG-3’; LINC00609: F 5’-TCCC ATCATTCTCGGCCCATCTC-3’, R 5’-TCTACTGCTCACACCTCCACTTCC-3’; LINC00634: F 5’-TGGTCATCTCGGTCCTC ATTCTGG-3’, R 5’-CCATTAGCGTCT TGCGGAGGTC-3’; POM121L9P F 5’-CGAAATGCCATCTCCAGCTCCTAC-3’, R 5’-GCCTGTCCTCACTCACTGTCTTT G-3’; SNHG11: F 5’-GCTGCCTTGGGTCTG GAAACTG-3’, R 5’-CATCACTCGTCACTCTTGGTCTGTG-3’; USP32P3: F 5’-CAG GCAAGGCAAAGGAAAATGACAG-3’, R 5’-CAGCCAGAGGATGACAGCAAGTG-3’; GAPDH: F 5’-GGATGATGTTCTGGAGAGCC-3’, R 5’-CATCACCATCTTCCAG GAGC-3’.

### Functional enrichment analysis and protein–protein interaction (PPI) network

Differential expression analysis was carried out to identify differential expressed genes between low- and high-risk subgroups of GBM patients from CGGA-GBM. R package “ClusterProfiler” was applied to perform Gene Ontology (GO) and Kyoto Encyclopedia of Genes and Genome (KEGG) enrichment analyses for upregulated and downregulated genes in high-risk subgroup respectively, with adj. *P* < *0.05* as the criteria of statistical significance^18,19^. Amongst, Gene Ontology (GO) terms consist of biological process (BP), cellular component (CC), molecular function (MF). According to the somatic mutation data of TCGA-GBM, the single-nucleotide variant (SNV) status of GBM samples in high-risk and low-risk subgroups were respectively extracted and profiled using the “Maftools” package of R. Besides, the PPI network of upregulated and downregulated genes in high-risk subgroup were respectively explored using version 11 of STRING (https://string-db.org/). The confidence score ≥ 0.4 was considered as the threshold and protein nodes without interaction with other proteins were removed. The PPI network was visualized by Cytoscape 3.7.2.

### Evaluation the correlation of the prognostic signature and immune activities

Single-sample gene set enrichment analysis (ssGSEA) was used to evaluate the infiltrating score of 16 types of immune cells and 13 types of immune-related functions based on the R package of ‘GSVA’. The infiltrating score of immune cells and immune-related functions were compared between low- and high-risk subgroups through Wilcoxon test, with *P* < 0.05 as statistically significant. The heatmap was drawn by “pheatmap” R package to display the differential immune activities.

## Results

### Identification of EMT-related lncRNAs in GBM

**Figure 1:**
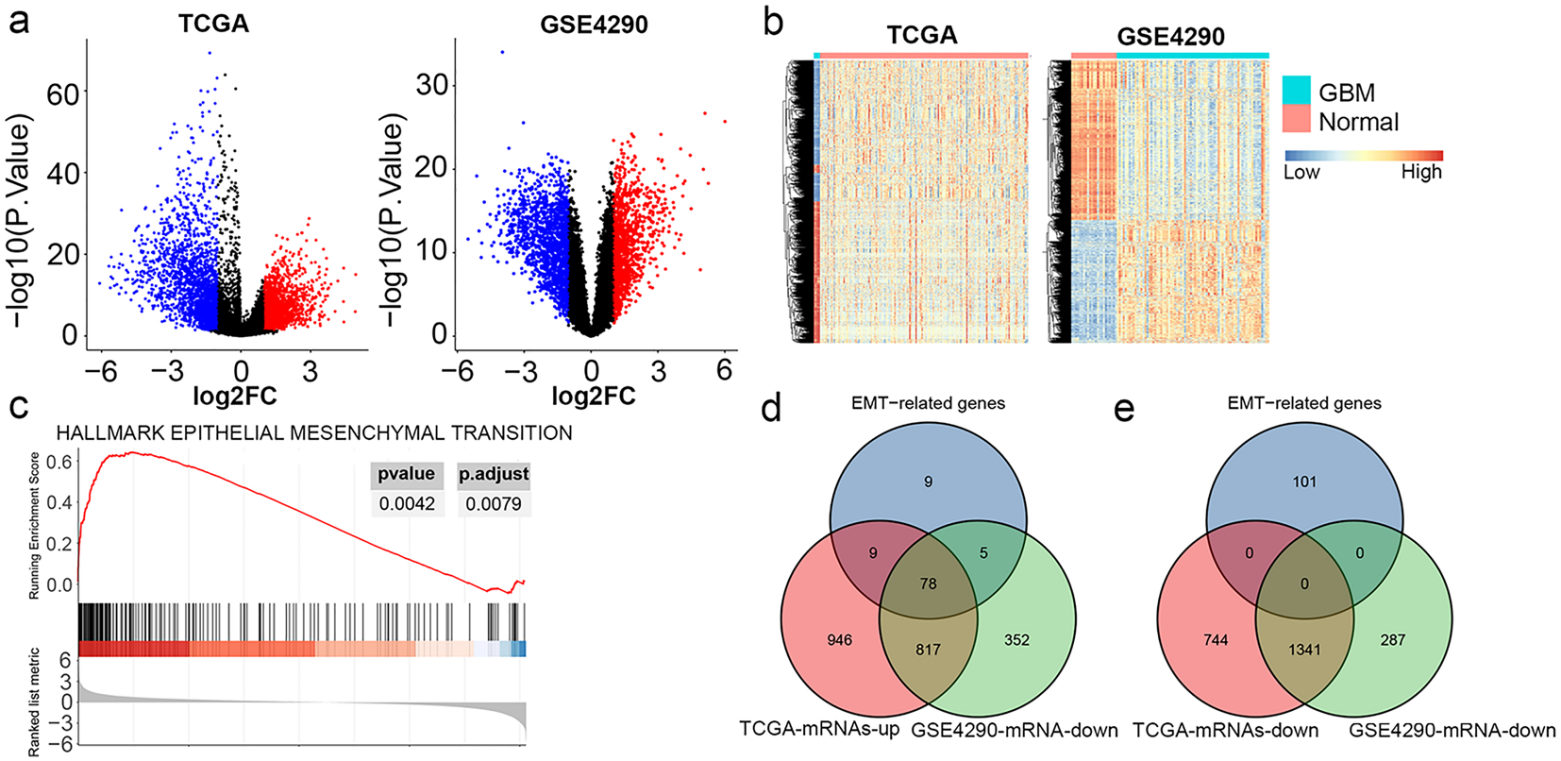
Identification of dysregulated EMT-related genes in GBM. (**a**) Volcano plots presenting the differences of mRNAs in GBM compared to normal tissues from TCGA and GSE4290 datasets. Blue dots represent downregulated mRNAs (log2FC < 1 and adj. *P* < 0.05); red dots represent upregulated mRNAs (log2FC > 1 and adj. *P* < 0.05). (**b**) Heatmaps of differentially expressed mRNAs between normal and GBM tissues from TCGA and GSE4290 datasets (|log2FC|≥ 0.5, adjusted *P* < 0.05). (**c**) GSEA plot showed the hallmark of epithelial-mesenchymal transition (EMT) was significantly enriched in GBM tissues (adj. *P* < 0.05). Venn diagrams showing 78 upregulated EMT-related genes (**d**) and 0 downregulated EMT-related genes (**e**) in GBM.

From TCGA-GBM, the expression of 9820 lncRNAs and 19641 mRNAs of 5 normal tissues and 156 GBM tissues were profiled. Through differential expression analysis, we identified 229 differentially expressed 3935 mRNA differentially expressed in GBM from TCGA (|log2FC|> 1 and adj. *P* < 0.05), among which 1850 were significantly upregulated while 2085 were significantly downregulated in GBM tissues. From GSE4290, a total of 16426 mRNA expression data of 23 normal tissues and 77 GBM tissues were collected for differential expression analysis, which identified 1252 upregulated and 1629 downregulated mRNAs in GBM tissues. The distribution of dysregulated mRNAs in GBM (TCGA-GBM and GSE4290) were shown in volcano plot (Fig. 1a). The heatmap of dysregulated mRNAs in GBM (TCGA-GBM and GSE4290) both obviously discriminate GBM tissues from normal tissues (Fig. 1b).

A total of 50 hallmark gene sets derived by concentrating multiple MSigDB gene sets, which represent well-defined biological states or processes, were selected for GSEA to explore differentially enriched hallmarks between 23 normal tissues and 77 GBM tissues from GSE4290. The results of GSEA identified 27 hallmarks significantly enriched in GBM tissues, with enrichment score > 1; while 2 hallmarks significantly enriched in normal tissues, with enrichment score < 1 (Supplementary Table S1). Amongst, EMT enriched 195 genes and got the enrichment score of 0.69 (Fig. 1c). Moreover, overlapping genes enriched in EMT from GSEA results, upregulated genes in GBM from TCGA, and upregulated genes in GBM from GSE4290, 78 genes were identified to be upregulated EMT-related genes in GBM (Fig. 1d, Supplementary Table S2). However, no EMT-related mRNA was downregulated in GBM (Fig. 1e). These results fully illustrated activation of EMT by EMT-related genes was one dominant mechanism of GBM malignancy. Furthermore, we aimed to explore EMT-related lncRNAs in the following. Through Spearman correlation analysis, we analyzed the correlations of lncRNAs and 78 upregulated EMT-related genes in GBM. According to the criteria described in Materials and Methods, a total of 301 lncRNAs were significantly correlated with EMT-related genes, which were considered to be EMT-related lncRNAs in GBM. The detailed relationships between EMT-related lncRNAs and genes were listed in Supplementary Table S3. Collectively, we confirmed EMT as one of determinants of GBM development and identified EMT-related lncRNAs in GBM.

### Construction of the prognostic signature consisting of 7 EMT-related lncRNAs for GBM patients

Firstly, we analyzed the correlations of EMT-related lncRNAs with OS of GBM patients from TCGA-GBM using univariable Cox regression. A total of 51 EMT-related lncRNAs were identified to be potential predictors of OS of GBM patients, among which 34 lncRNAs with HR > 1, while 17 lncRNAs with HR < 1 (Table 1). Then, to construct a credible prognostic model for GBM patients based on EMT-related lncRNAs, TCGA-GBM cohort and CGGA-GBM cohort were used as training set and validating set, respectively. Therefore, 21 of 51 prognostic EMT-related lncRNAs annotated both in TCGA-GBM and CGGA-GBM were selected for the following multivariate Cox regression analysis. As a result, 7 EMT-related lncRNAs with non-zero coefficients (AC012615.1, H19, LINC00609, LINC00634, POM121L9P, SNHG11, and USP32P3) were determined and their corresponding coefficients were listed in Table 2. Accordingly, the risk score of GBM patients could be calculated by the following formula: Risk score = AC012615.1 expression × (− 0.453878786202157) + LINC00957 expression × 0.0828358815945787 + LINC02532 expression × 0.355210689402953 + LINC00634 × (− 0.220300418653349) + POM121L9P expression × 0.376871997524452 + SNHG11 × 0.598418567613137 + USP32P3 expression ×  (− 0.284320135850659). Taken together, the EMT-related lncRNA prognostic signature composed of AC012615.1, H19, LINC00609, LINC00634, POM121L9P, SNHG11, and USP32P3 was established for GBM patients through univariate and multivariate Cox regression analyses.

**Table 1:** Detailed information of 51 EMT-related lncRNAs significantly associated with OS of GBM patients.

EMT-related lncRNAs	HR	95% lower HR	95% higher HR	*P* value
AL645608.6	0.650555	0.45191	0.936518	0.020735
TP73-AS1	1.67561	1.248667	2.248532	0.000582
AC105277.1	1.472884	1.053052	2.060094	0.023702
AC099792.1	0.620568	0.442426	0.870439	0.005715
FCGR2C	1.370341	1.059648	1.77213	0.016324
CRYZL2P	1.397479	1.019386	1.915807	0.037594
AC104794.2	1.763515	1.24148	2.505061	0.001536
CYTOR	1.240667	1.021612	1.506692	0.029579
LINC01614	1.43594	1.12055	1.840101	0.004243
AC131097.4	1.404002	1.014985	1.942121	0.040383
SNHG18	1.239572	1.021453	1.504268	0.029636
HLA-J	1.438985	1.022885	2.024352	0.036627
AC092171.2	1.422939	1.050528	1.927368	0.022705
LINC00957	1.386648	1.07941	1.781337	0.01053
SBDSP1	1.564308	1.120248	2.184391	0.008627
LINC00689	0.867517	0.752987	0.999468	0.049145
MIR222HG	1.788026	1.256503	2.544392	0.001244
AL157700.1	0.620692	0.452137	0.852084	0.003176
LINC01605	1.401736	1.057968	1.857205	0.018648
AL512625.2	0.722176	0.539402	0.966882	0.028803
LINC00092	1.444684	1.121957	1.860241	0.004343
LINC01503	1.307611	1.08304	1.578748	0.005275
H19	1.097872	1.003513	1.201104	0.041705
AC009549.1	1.250691	1.038081	1.506846	0.018617
SNHG1	0.731428	0.539982	0.990748	0.043384
LINC00836	0.64885	0.480179	0.876769	0.00486
LINC00941	1.345768	1.038935	1.74322	0.024496
RPS27P25	1.483728	1.059459	2.077898	0.021671
SOX21-AS1	0.628814	0.469472	0.842237	0.001861
AL133304.3	0.739083	0.563591	0.969219	0.028815
LINC00609	0.714254	0.51264	0.99516	0.046741
AC004816.2	1.508116	1.062599	2.140425	0.021457
AC013391.3	0.751462	0.610926	0.924326	0.006833
MIR22HG	1.379092	1.060873	1.792764	0.01633
AC015922.2	1.253088	1.043146	1.505284	0.015889
AC015922.3	1.342042	1.092209	1.649023	0.005123
USP32P3	0.757714	0.590385	0.972466	0.029312
AC002401.4	1.333968	1.082619	1.643672	0.006826
AC061992.1	1.386256	1.05911	1.814455	0.017401
AC110285.1	0.818067	0.669505	0.999596	0.04954
AL118505.1	0.720886	0.581854	0.893139	0.002756
SNHG11	1.673999	1.212466	2.311218	0.001745
SMIM25	1.304072	1.040159	1.634944	0.021377
BX640514.2	1.326605	1.013488	1.736461	0.039638
AC012615.1	0.727533	0.557015	0.950252	0.019572
ZNF528-AS1	0.731075	0.537022	0.995248	0.046566
ZDHHC8P1	1.402816	1.072085	1.835574	0.013612
POM121L9P	1.690536	1.218279	2.34586	0.001683
LINC00634	0.779214	0.618678	0.981405	0.034054
MIR155HG	1.337961	1.035838	1.728206	0.025774
BX322562.1	1.425814	1.045585	1.944314	0.024984

*EMT* epithelial-mesenchymal transition, *lncRNAs* long non-coding RNAs, *OS* overall survival, *GBM* glioblastoma, *HR* hazard ratio.

**Table 2:** The final prognostic risk score model for GBM patients.

EMT-related lncRNA	HR	95% lower HR	95% higher HR	*P* value	Coefficient
AC012615.1	0.63516	0.471687	0.855288	0.002794	− 0.45388
H19	1.086364	0.986396	1.196463	0.092597	0.082836
LINC00609	1.426481	0.921887	2.207265	0.110757	0.355211
LINC00634	0.802278	0.61518	1.046278	0.103939	− 0.2203
POM121L9P	1.457718	1.012034	2.099673	0.042948	0.376872
SNHG11	1.81924	1.229622	2.691585	0.002751	0.598419
USP32P3	0.752526	0.555687	1.01909	0.066101	− 0.28432

*EMT* epithelial-mesenchymal transition, *GBM* Glioblastoma, *HR* hazard ratio.

### Prognostic value of the EMT-related lncRNA signature

**Figure 2:**
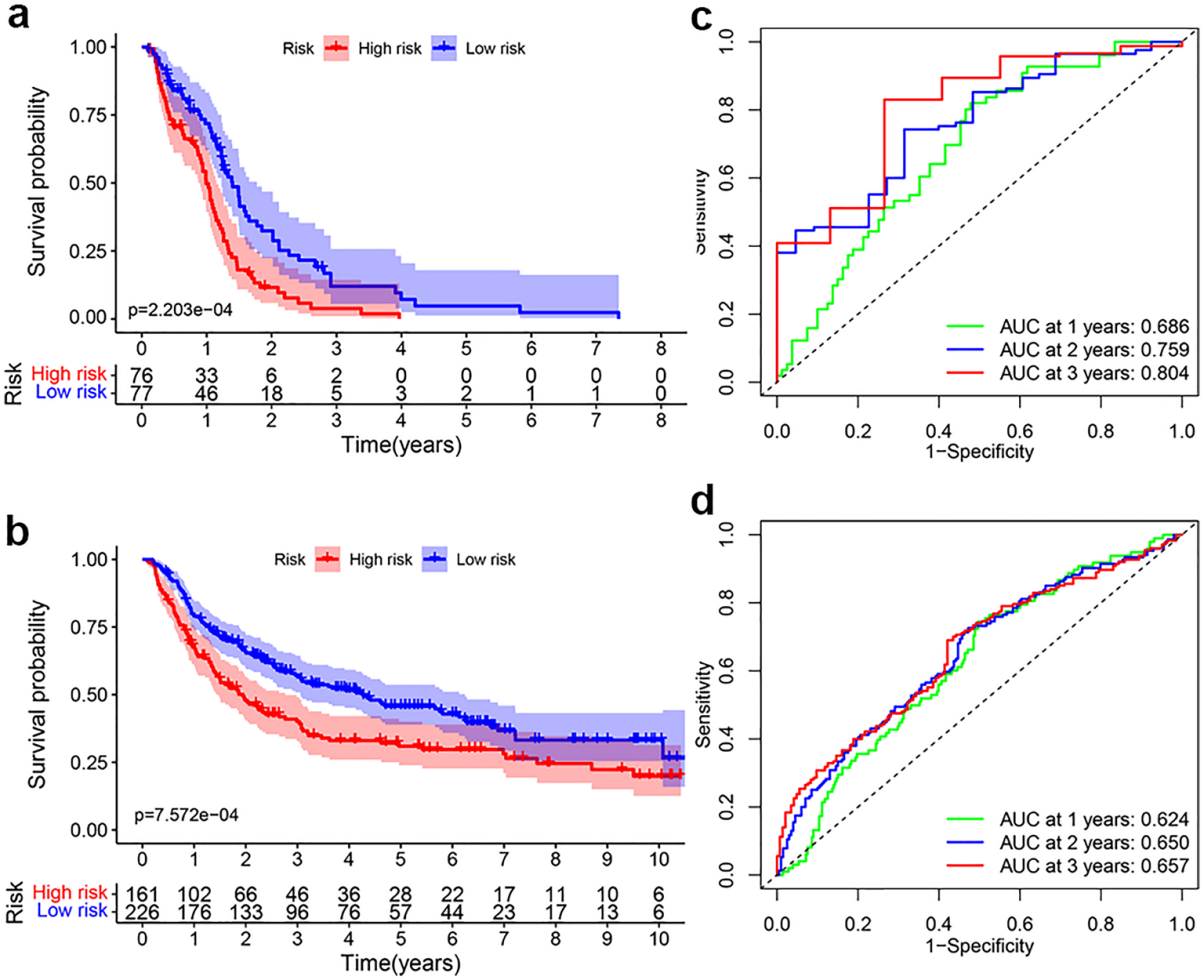
Validating the accuracy and efficiency of the EMT-related lncRNA prognostic signature. Kaplan–Meier analysis of the prognostic signature for GBM patients in training set (**a**) and validating set (**b**), respectively. ROC curve for GBM patients in training set (**c**) and validating set (**d**), respectively.

**Figure 3:**
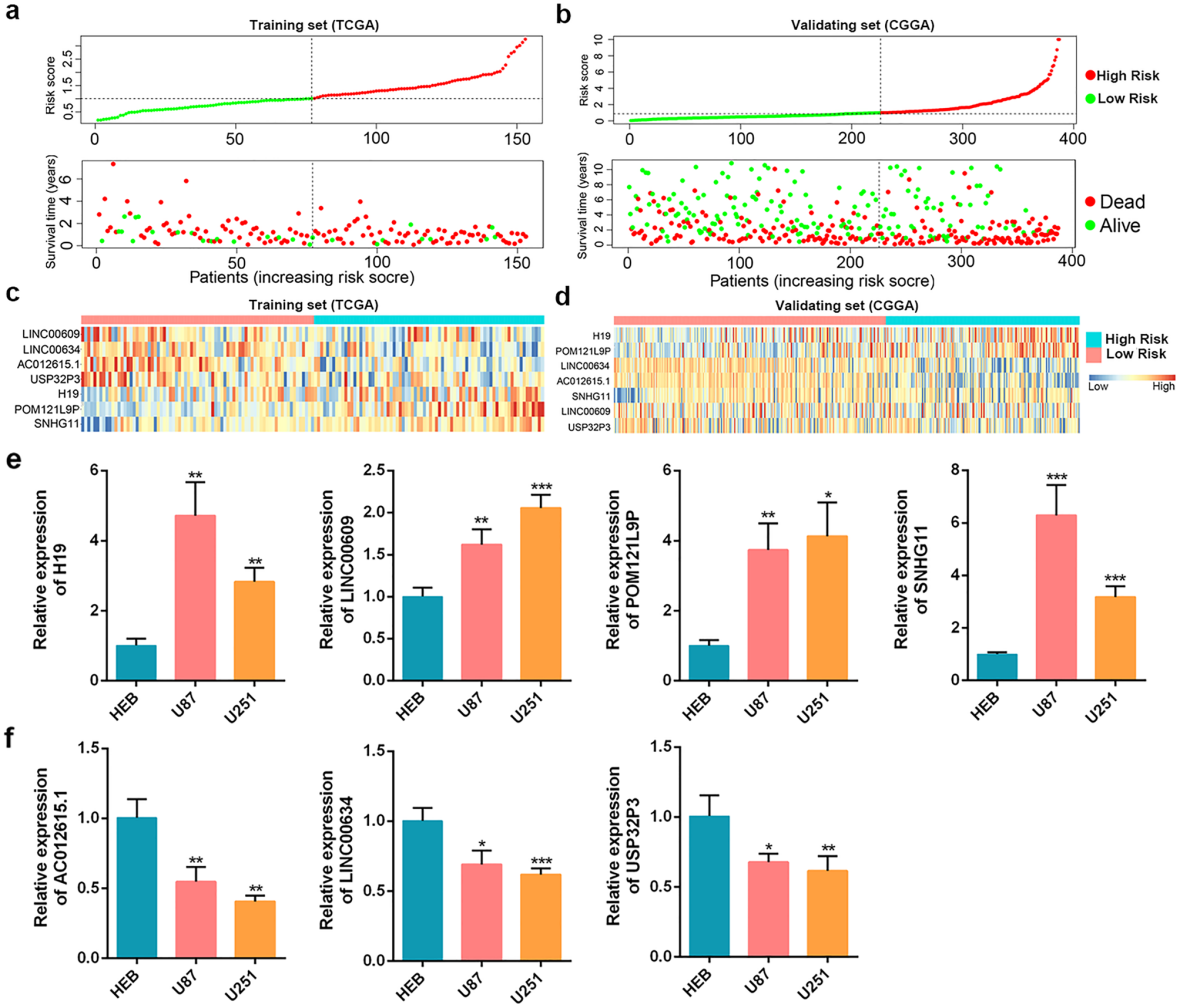
The distribution of risk scores, survival status, and expression patterns of EMT-related lncRNAs in the prognostic signature. The distribution of risk score (upper) and survival status (lower) of GBM patients in training set (**a**) and validating set (**b**). Heatmap displaying expression patterns of the seven EMT-related lncRNAs in GBM patients of training set (**c**) and validating set (**d**). (**e**) The relative expression levels of H19, LINC00609, POM121L9P, and SNHG11 in normal human glial cell line HEB and glioma cell lines (U87 and) U251 were measured by qRT-PCR. (**f**) The relative expression levels of AC012615.1, LINC00634, and USP32P3 in normal human glial cell line HEB and glioma cell lines (U87 and) U251 were detected by qRT-PCR. *P < 0.05, **P < 0.01, ***P < 0.001.

To evaluate the prognostic ability of the EMT-related lncRNA signature, the risk scores of each GBM patients in training set and validating set were calculated by the formula mentioned above. With the median value of risk scores in training set (1.006382) as cut-off value, GBM patients of training set and validating set were respectively divided into low- and high-risk subgroups. The Kaplan–Meier survival curves showed GBM patients of low-risk subgroup had better prognosis compared with those of high-risk subgroup in both the training and testing sets (Fig. 2a,b). ROC curve analysis revealed the AUC values of 1-, 3-, and 5-year were 0.686, 0.804 and 0.883 in the training set, and 0.624, 0.657, and 0.641 in the validating set, respectively (Fig. 2c,d). Additionally, the risk score plot, survival time and status plot for training set and validating set both demonstrated reduced survival time and more death events in high-risk subgroup compared with those in low-risk subgroup (Fig. 3a,b). The heatmap showed significantly differential expression of AC012615.1, H19, LINC00609, LINC00634, POM121L9P, SNHG11, and USP32P3 between the low- and high-risk subgroups (Fig. 3c,d). Importantly, we validated H19, LINC00609, POM121L9P, and SNHG11, which as risk factors of GBM, were significantly upregulated; while AC012615.1, LINC00634, and USP32P3, which as protective factors of GBM, were dramatically decreased in glioma cell lines (U87 and U251), compared to Normal human glial cell line HEB (Fig. 3e,f). Altogether, these results indicated the high accuracy and efficiency of our prognostic signature.

### Multiple EMT and metastasis-related pathways are responsible for the risk of GBM patients based on the EMT-related lncRNA signature

**Figure 4:**
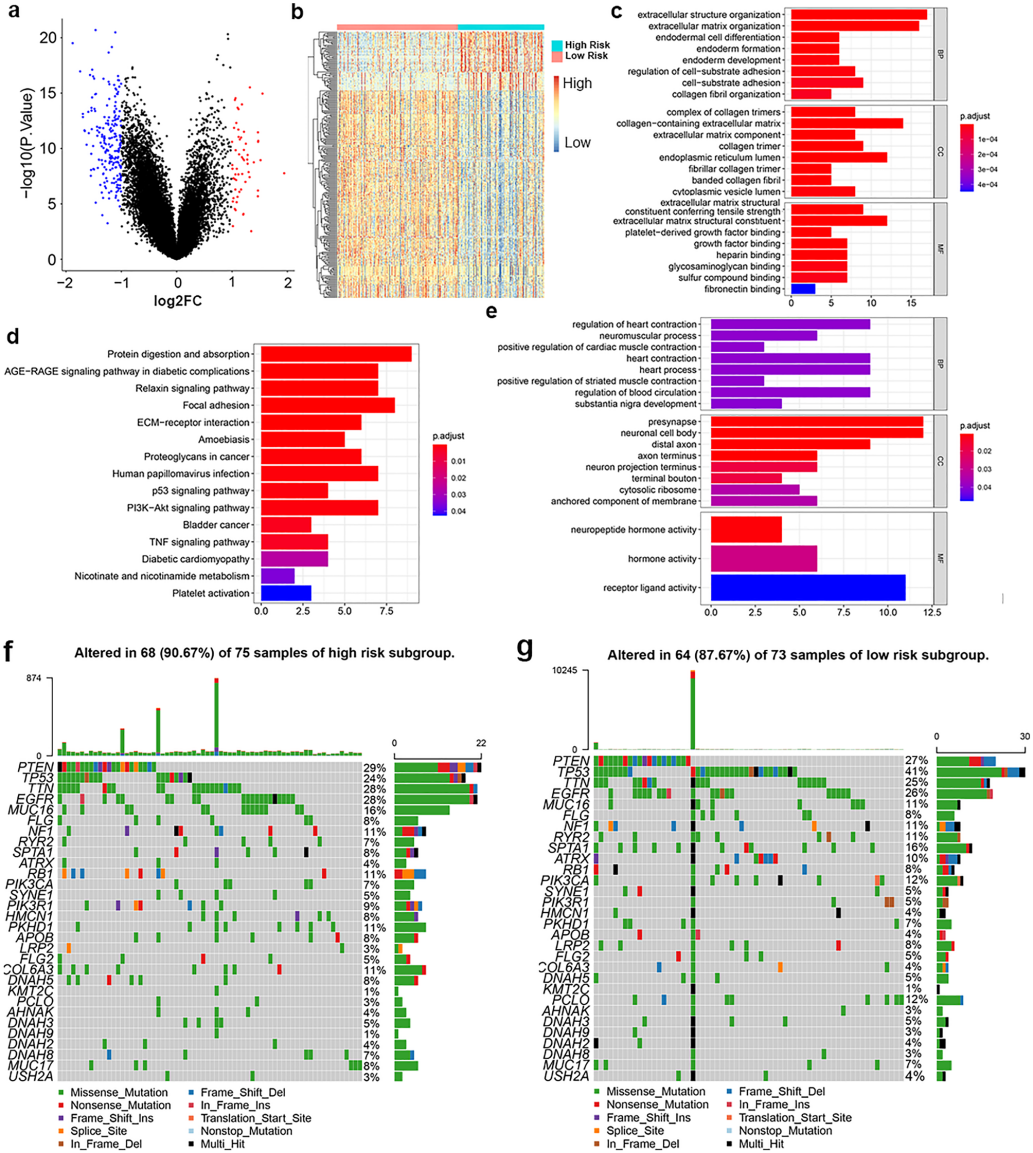
Exploration of the underlying mechanisms of the prognostic signature. (**a**) Volcano plot displaying the differentially expressed genes between GBM patients of low-risk and high-risk subgroups. Blue dots represent downregulated genes in high-risk subgroup (log2FC < 1 and adj. *P* < 0.05); red dots represent upregulated genes in high-risk subgroup (log2FC > 1 and adj. *P* < 0.05). (**b**) Heatmaps of differentially expressed genes between GBM patients of low-risk and high-risk subgroups (|log2FC|≥ 0.5, adjusted *P* < 0.05). **(c)** GO analysis for upregulated genes in GBM patients of high-risk subgroup. Top terms of BP, CC, and MF with adj. *P* < 0.05 were displayed. (d) KEGG analysis for upregulated genes in GBM patients of high-risk subgroup. Top pathways with adj. *P* < 0.05 were shown. (**e**) GO analysis for downregulated genes in GBM patients of high-risk subgroup. Top terms of BP, CC, and MF with adj. *P* < 0.05 were displayed. (**f**,**g**) Waterfall maps of top 30 mutated genes in high-/low-risk subgroups.

**Figure 5:**
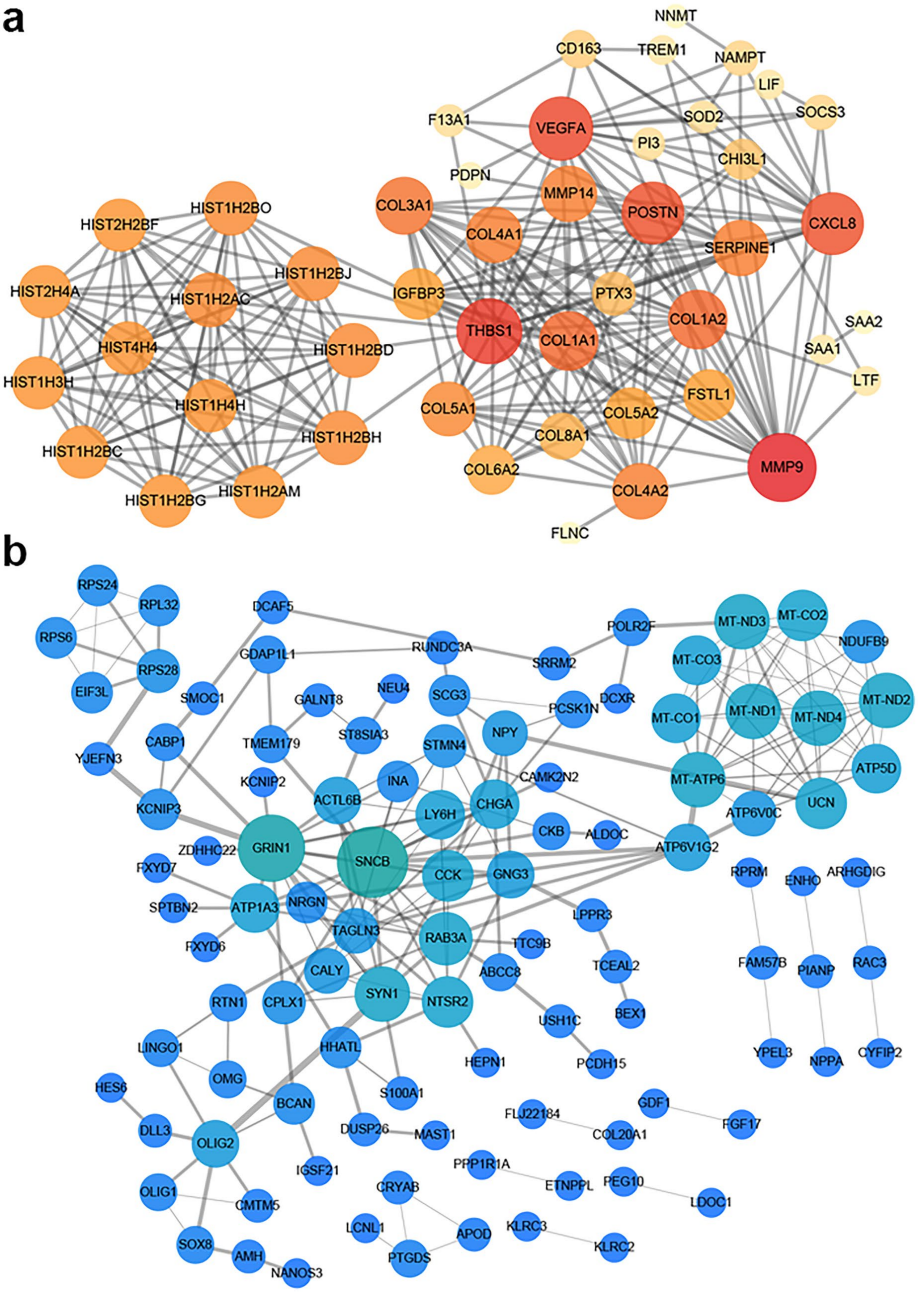
PPI network construction for dysregulated genes in GBM patients of high-risk subgroup. (**a**) PPI network of upregulated genes in GBM patients of high-risk subgroup. (**b**) PPI network of downregulated genes in GBM patients of high-risk subgroup.

It is meaningful to explore the biological functions associated with the EMT-related lncRNA signature to understand its clinical value in guiding therapy. To further elucidate differential biological features between low- and high-risk subgroups of GBM patients, differential expression analysis was performed and identified 268 differentially expressed genes (|log2FC|> 1 and adj. *P* < 0.05), among which 59 genes were upregulated and 209 genes were downregulated in high-risk subgroup. The distribution of dysregulated genes was shown in volcano plot (Fig. 4a). The heatmap of dysregulated genes was displayed in Fig. 4b. Following GO functional annotation and KEGG pathway enrichment analysis were applied for the 59 upregulated genes and 209 downregulated genes respectively. In GO, upregulated genes involved in organization of extracellular structure and matrix, endodermal cell differentiation, endoderm formation and organization, regulation of cell-substrate adhesion in BP; complex of collagen trimers, collagen-containing extracellular matrix, and extracellular matrix component in CC; extracellular matrix structural constituent, platelet-derived growth factor binding, heparin binding in MF. (Fig. 4c). In KEGG, upregulated genes were significantly enriched in Focal adhesion, ECM-receptor interaction, and PI3K-Akt signaling pathway, etc. (Fig. 4d). Moreover, downregulated genes involved in regulation of cardiac muscle contraction and striated muscle contraction, neuromuscular process, regulation of blood circulation, and substantia nigra development in BP; presynapse, neuronal cell body, and distal axon in CC; neuropeptide hormone activity, hormone activity, and receptor ligand activity in MF (Fig. 4e). However, no pathway was found to be associated with downregulated genes. The detailed results of GO and KEGG analyses were provided in Supplementary Table S4. Then we profiled the SNV distribution of 75 GBM samples in high-risk subgroup and 73 GBM samples in low-risk subgroup, respectively (Fig. 4f,g). Amongst, PTEN, TP53, TTN, and EGFR exhibited high rates of SNV in both high risk and low risk subgroups. Significantly, the SNV rates of PTEN, TTN, EGFR, MUC16, NF1, RB1, PIK3R1, HMCN1, PKHD1, APOB, COL6A3, and DNAH5 were higher in high-risk subgroup; while TP53, RYR2, SPTA1, ATRX, PIK3CA, LRP2, and PCLO showed higher rates of SNV in low-risk subgroup. These different SNV distribution might be responsible for the risk of GBM patients to some extent. Moreover, the PPI network of upregulated genes and downregulated genes were respectively constructed using STRING and visualized by Cytoscape, demonstrating the complex intersections of these genes (Fig. 5a,b). Collectively, these results revealed the essential roles of genes upregulated in high-risk subgroup in the EMT and metastasis of GBM, while genes downregulated in high-risk subgroup mainly participate in various important physiological processes.

### Identification the correlation of the prognostic signature and immune activities in GBM

**Figure 6:**
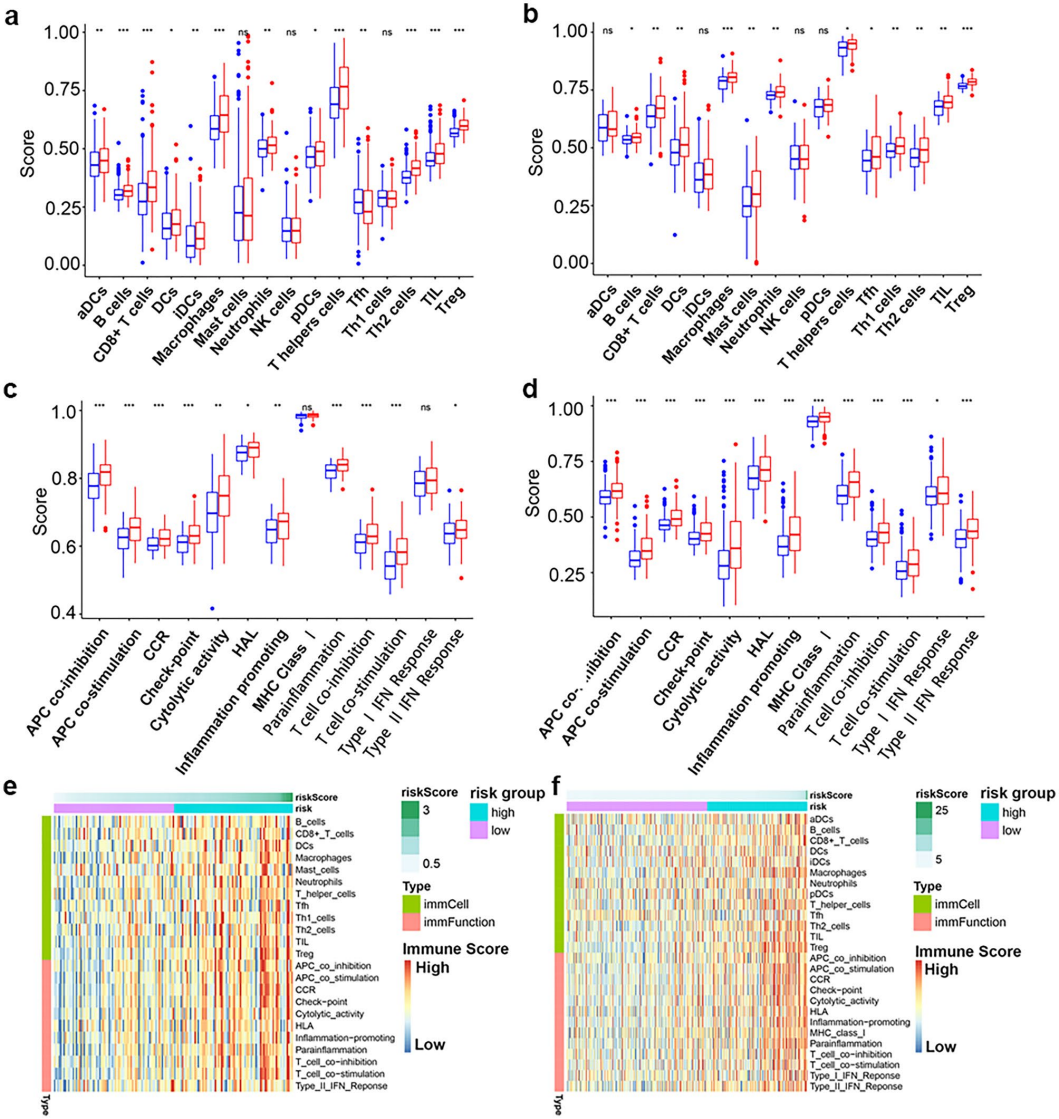
The EMT-related lncRNA signature significantly associated with immune activities of GBM. Comparison of the abundance of 16 immune cells types between low-risk and high-risk subgroups from TCGA (**a**) and CGGA (**b**). Comparison of the abundance of 13 immune response types between low-risk and high-risk subgroups from TCGA (**c**) and CGGA (**d**). Heatmap showing the differential immune activities between low-risk and high-risk subgroups from TCGA (**e**) and CGGA (**f**).

Considering the correlation of immune infiltration status and EMT phenotype of tumors, it is worthwhile to explore the relationship between the prognostic signature and immune activities of GBM. Therefore, we employed single sample GSEA (ssGSEA) to acquire the abundance of 16 immune cell types and 13 immune response types of GBM patients from TCGA-GBM and CGGA-GBM, respectively. For TCGA-GBM cohort, the abundance of 12 immune cells types and 11 immune response types showed a significant increase in GBM patients in high-risk subgroup compared to those in the high-risk subgroup (Fig. 6a,b). Similarly, GBM patients of high-risk subgroup showed a dramatically higher abundance of 13 immune cells types and 13 immune response types than those in low-risk subgroup (Fig. 6c,d). Besides, the heatmaps of differential immune activities in TCGA-GBM and CGGA-GBM cohorts were visualized in Fig. 6e,f, respectively. These results confirmed that the infiltration of immune cells and immune responses in glioma were significantly increased in GBM patients of high-risk subgroup.

## Discussion

Glioblastoma (GBM) is the most common and aggressive primary brain malignancy in adults^20^. Due to the high infiltration and heterogeneity of GBM, it is a great challenge to improve its poor prognosis, low survival and high recurrence rates. With the advancement of microarray and sequencing technologies, increasing lncRNAs were confirmed to have potential to be novel and effective diagnostic, therapeutic and prognostic targets for GBM patients. For example, Tian et al. have reported lncRNA AGAP2-AS1 is significantly upregulated in GBM and high expression levels of AGAP2-AS1 predicts is a marker of poor prognosis for GBM patients^21^. Generally, the initiation and progression of GBM is orchestrated by multiple factors^22^. Therefore, the capacity of a single gene to predict the prognosis is extremely limited. Currently, increasing studies dedicated to develop prognostic signatures composed of various tumor hallmarks-related lncRNAs, providing promising prognostic indicators and personalized treatments for tumor malignancies. Wang et al. established a valuable glycolysis-related lncRNA signature to predict the prognosis of diffuse glioma patients through univariate Cox regression analysis, the Least Absolute Shrinkage and Selection Operator (LASSO) analysis and multivariate Cox regression analysis^23^. Luan et al. constructed a prognostic signature composed of 10 autophagy-related lncRNAs for glioma patients^24^.

In our present study, we firstly explored the key mechanisms responsible for the development of GBM and found out 29 hallmarks exhibited significant different enrichment between non-tumor and GBM tissues. Among these 29 hallmarks, EMT, angiogenesis, glycolysis, hypoxia have been confirmed to be critical processes of cancer progression and metastasis. More importantly, 78 of 195 genes enriched in EMT were upregulated both in GBM patients from TCGA and GSE4290, which further proving the close link between EMT and GBM development. A significant number of lncRNAs have proved to be essential regulators of EMT^25^. In our present study, we analyzed the correlation of lncRNAs and EMT-related mRNAs in GBM and identified 301 EMT-related lncRNAs. Among these 301 EMT-related lncRNAs, LINC00511 and NEAT1 have been reported to promote malignancy of GBM through EMT; while lncRNA DGCR5 was demonstrated to suppress the migration, invasiveness of GBM cells through reversing EMT process^26–28^. In our study, LINC00511 and NEAT1 were positively correlated with upregulated EMT-related mRNAs, while DGCR5 was negatively correlated with upregulated EMT-related mRNAs, suggesting the potential of LINC00511 and NEAT to promote EMT, DGCR5 to reverse EMT process. The consistency of our results and existing studies proved the reliability of our study. However, the regulatory roles and corresponding molecular mechanisms of the majority of these 301 EMT-related lncRNA in EMT of GBM have not been explored, which need substantial work to be carried out in future.

Several studies have considered the prognostic value of EMT-related lncRNAs for tumors. Xiao et al. constructed an eight EMT-related lncRNA signature for melanoma to predict individualized prognosis and therapeutic effects^29^. Du et al. identified a five stromal EMT-related lncRNA for bladder cancer, which could be used to predict the prognosis and responsiveness to immune checkpoint blockade therapy^30^. Zhang et al. established a survival signature based on EMT-related differentially expressed lncRNAs for patients with kidney renal clear cell carcinoma^31^. It is worth mentioning that the all of these EMT-related lncRNA signature can provide accurate and effective prediction for patients with their corresponding cancer types, indicating considerable clinical value of EMT-related lncRNAs for cancers. However, few studies have focused on the relationship between EMT-related lncRNA and the prognosis of GBM patients. Here, we analyzed the prognostic value of EMT-related lncRNAs in GBM through univariate Cox regression analysis, and constructed an EMT-related lncRNAs prognostic signature for GBM through multivariate Cox regression analysis. Then the prognostic value and robustness of the EMT-related lncRNA signature were validated through Kaplan–Meier survival analysis and ROC curve analysis. Recently, multiple prognostic models have been proposed for patients with GBM. The AUC values of a five-lncRNAs signature established by Niu et al. reached 0.690, 0.704, and 0.709 for 1-, 2-, and 3-year survival, respectively. Besides, the AUC values of a 5 immune-related lncRNA risk signature constructed by Li et al. were 0.671 to predict 1-year survival and 0.809 to predict 2-year survival^32^. Compared with above published signatures, the AUC values of our EMT-related lncRNAs signature are relatively satisfactory both in training set and validating set.

Among the seven EMT-related lncRNAs in the prognostic model, H19, LINC00609, POM121L9P, and SNHG11 were risk factors of GBM, while AC012615.1, LINC00634, and USP32P3 were protective factors for GBM. To date, the oncogenic roles and molecular mechanisms of H19, a maternally imprinted gene, have been extensively studies in multiple cancers, including glioma, breast cancer, and lung cancer, etc.^33–35^. H19 participates in regulation of angiogenesis, autophagy, as well as cell proliferation, migration and invasion^36^. POM121L9P, located on chromosome 22 (22q11.23), has been reported to be associated with shorter OS and poor clinicopathological features of epithelial ovarian cancer^37^. SNHG11, a small nucleolar RNA (snoRNA) host gene, is a well-recognized cancer-promoting lncRNA and promotes autophagy, proliferation, migration, and invasion of multiple tumor cells. Of note, the promoting effect of H19 and SNHG11 on EMT have been validated in gastric cancer, hepatocellular cancer, and glioma^38–40^. Consistently, our study showed H19 and SNHG11 were oncogenes and positively correlated with upregulated EMT-related genes in GBM, indicating their potential facilitative roles in regulating the EMT of GBM. Zhang et al. have reported LINC00634 is upregulated in esophageal cancer and functions as an oncogene through miR-342-3p/Bcl2L1 axis to promote cell viability and inhibit cell apoptosis^41^. Paradoxically, our study suggested LINC00634 was a tumor suppressor and negatively associated with upregulated EMT-related genes, suggesting the inhibitory roles of LINC00634 in EMT of GBM. It is possible that a gene plays opposite roles in different cancers^42^. Admittedly, regardless of whether the results are consistent or contrary to previous studies about other cancer types, further experimental verification is required. More importantly, no study has reported the roles of LINC00609, AC012615.1, and USP32P3 in cancers. Therefore, even the prognostic performance of the seven EMT-related lncRNAs signature in this study is excellent, the specific regulatory roles and underlying mechanisms of all these seven lncRNAs in EMT of GBM need to be further investigated in-depth.

GO functional annotation and KEGG pathway enrichment analyses showed high-risk scores based on our EMT-related lncRNA signature heralded the EMT process and metastatic phenotype of GBM, which explained why the GBM patients in high-risk subgroup had worse prognosis. Indeed, some studies have elaborated that EMT phenotype confers cancers to be more sensitive to immune targeting strategies whereas others have linked EMT phenotype with immunotherapy resistance^43,44^. Therefore, it is possible that distinct immune infiltration pattern among GBM patients with different risk scores based the EMT-related lncRNA signature. Currently, immune checkpoint blockade is expected to be one of the next frontiers in cancer immunotherapy^45^. We compared the infiltration of immune cells and immune responses between low- and high-risk subgroups. As a result, degrees of immune cell infiltration and immune responses were largely increased in GBM patients of high‐risk subgroup, indicating that even though GBM patients of high-risk subgroup with worse prognosis, their response to immune checkpoint blockade therapies may be batter.

There were several limitations in the present study: Firstly, our EMT-related lncRNA prognostic signature was constructed and validated based on the public dataset, which requires more prospective clinical data for clinical application in future. Secondly, the EMT-related lncRNAs in GBM were identified based on their expression correlation with EMT-related mRNA. Even though several lncRNAs have been validated to participate in regulation of EMT, their roles and molecular functions in GBM need to be further explored through in vivo and in vitro experiments. Thirdly, we identified the correlation of the EMT-related lncRNA signature and immune activities in GBM, but the underlying mechanisms remains to be investigated in-depth in future studies.

## Conclusion

In conclusion, our present study highlighted the importance of EMT in GBM progression and identified EMT-related lncRNA in GBM, and constructed a seven EMT-related lncRNA prognostic signature for GBM with relatively high efficiency and accuracy. The EMT-related lncRNA prognostic signature was associated with EMT phenotype and immune infiltration status.

## Supplementary Information

Supplementary Table S1.

Supplementary Table S2.

Supplementary Table S3.

Supplementary Table S4.

## Data Availability

All data used by the study are publicly available in the TCGA-GBM (http://xena.ucsc.edu/), CGGA (http://www.cgga.org.cn), and GSE4290 (https://www.ncbi.nlm.nih.gov/geo/query/acc.cgi?acc=GSE4290).
